# Exceptional long-term survival in an adult with advanced classic biphasic pulmonary blastoma: a case report and literature review

**DOI:** 10.3389/fonc.2025.1727839

**Published:** 2026-01-15

**Authors:** Jun Li, Si Chen, Qiliang Liu, Ming Li

**Affiliations:** 1 Department of Oncology, Wuhan Pulmonary Hospital, Wuhan, Hubei, China; 2 Department of Pathology, Wuhan Pulmonary Hospital, Wuhan, Hubei, China

**Keywords:** classic biphasic pulmonary blastoma, long-term survival, multimodal therapy, neoadjuvant chemotherapy, prognosis, pulmonary blastoma, surgery

## Abstract

**Background:**

Pulmonary blastoma (PB) is an exceedingly rare and aggressive primary lung malignancy. Classic biphasic pulmonary blastoma (CBPB), a subtype, is associated with a historically poor prognosis, particularly in advanced stages. There is no standard treatment for advanced CBPB, and reports of long-term survival are exceptionally rare. We present a case of prolonged disease-free survival in an adult with stage IIIC CBPB and review contemporary long-term survivors to identify prognostic factors.

**Case presentation:**

A 32-year-old female presented with cough and dyspnea. Imaging revealed a large mass in the left upper lobe with mediastinal and supraclavicular lymph node involvement, leading to a diagnosis of stage IIIC CBPB. The patient received six cycles of neoadjuvant chemotherapy with ifosfamide plus epirubicin (IFO + EPI) and achieved a partial response. She subsequently underwent left upper lobectomy and lymph node dissection. Due to dense adhesion of mediastinal lymph node station 5 to the aortic arch, phrenic nerve, and vagus nerve, complete resection was not feasible, resulting in R2 resection (macroscopic residual disease). Postoperatively, she completed adjuvant chemoradiotherapy (one cycle of IFO + EPI, concurrent radiotherapy 54 Gy combined with docetaxel plus carboplatin, and two cycles of docetaxel plus cisplatin). As of July 2025, the patient has remained disease-free for more than six years post-diagnosis, demonstrating exceptional long-term survival.

**Conclusion:**

This case highlights that aggressive multimodal therapy, including neoadjuvant chemotherapy, surgical resection, and adjuvant radiotherapy, can lead to long-term survival in advanced CBPB patients. A literature review (from 2000 to present) revealed 13 CBPB patients surviving > 3 years, with 5 of them surviving ≥ 5 years. Most of these patients underwent surgery and multimodal treatment. Aggressive local therapy for recurrent disease and antiangiogenic agents and immunotherapy represent promising strategies to improve outcomes in this rare malignancy.

## Introduction

Pulmonary blastoma (PB) is an exceedingly rare primary malignant lung tumor, accounting for approximately 0.25%–0.5% of all pulmonary neoplasms (1–3). It is estimated that fewer than 100 new cases are diagnosed globally each year (4). Histologically, PB is composed of immature epithelial and mesenchymal elements, originates from the lung or pleura, and is characterized by high aggressiveness and poor prognosis (1–3). Among PB, classic biphasic pulmonary blastoma (CBPB) is a representative subtype defined by its typical biphasic morphology composed of both epithelial and mesenchymal components. The disease predominantly occurs in adults, with epidemiological data suggesting a slightly higher incidence in males and a certain association with smoking (5, 6). In historical classifications, CBPB was grouped with well-differentiated fetal adenocarcinoma and pleuropulmonary blastoma. According to the latest 2021 World Health Organization classification, CBPB has been clearly defined as an independent disease entity belonging to the category of pulmonary sarcomatoid carcinoma (7). Patient clinical manifestations often include chest pain, cough, and hemoptysis, with approximately one-third of cases being asymptomatic (8). Diagnosis relies on histopathological biopsy. Currently, complete surgical resection remains the primary potential curative approach. However, the overall prognosis of this disease is poor, with previous reports indicating a 5-year survival rate below 20% (2, 8) and a high risk of recurrence, often occurring early after surgery. Currently, there is no standardized treatment regimen for PB. Patients with early- and intermediate-stage disease typically receive comprehensive therapy centered on radical surgery. For advanced, unresectable cases, treatment options are limited, primarily relying on radiotherapy and systemic chemotherapy, but the survival benefit from conventional regimens is modest (9, 10).

Over the past two decades, with a deepening understanding of tumor biology and advancements in treatment modalities, the prognosis of PB has shown a trend of improvement. This paper reports an adult patient with stage IIIC CBPB who achieved clinical cure after comprehensive treatment, including neoadjuvant chemotherapy, radical surgery, and postoperative radiotherapy, maintaining disease-free survival for over six years as of the last follow-up in July 2025. Furthermore, through a systematic literature review, we included 13 CBPB patients reported between 2000 and 2025 with survival exceeding 3 years, aiming to analyze their clinical characteristics and treatment patterns, summarize treatment strategies associated with long-term survival in this disease, and provide references for the clinical management of this rare malignancy.

## Case report

**Figure 1:**
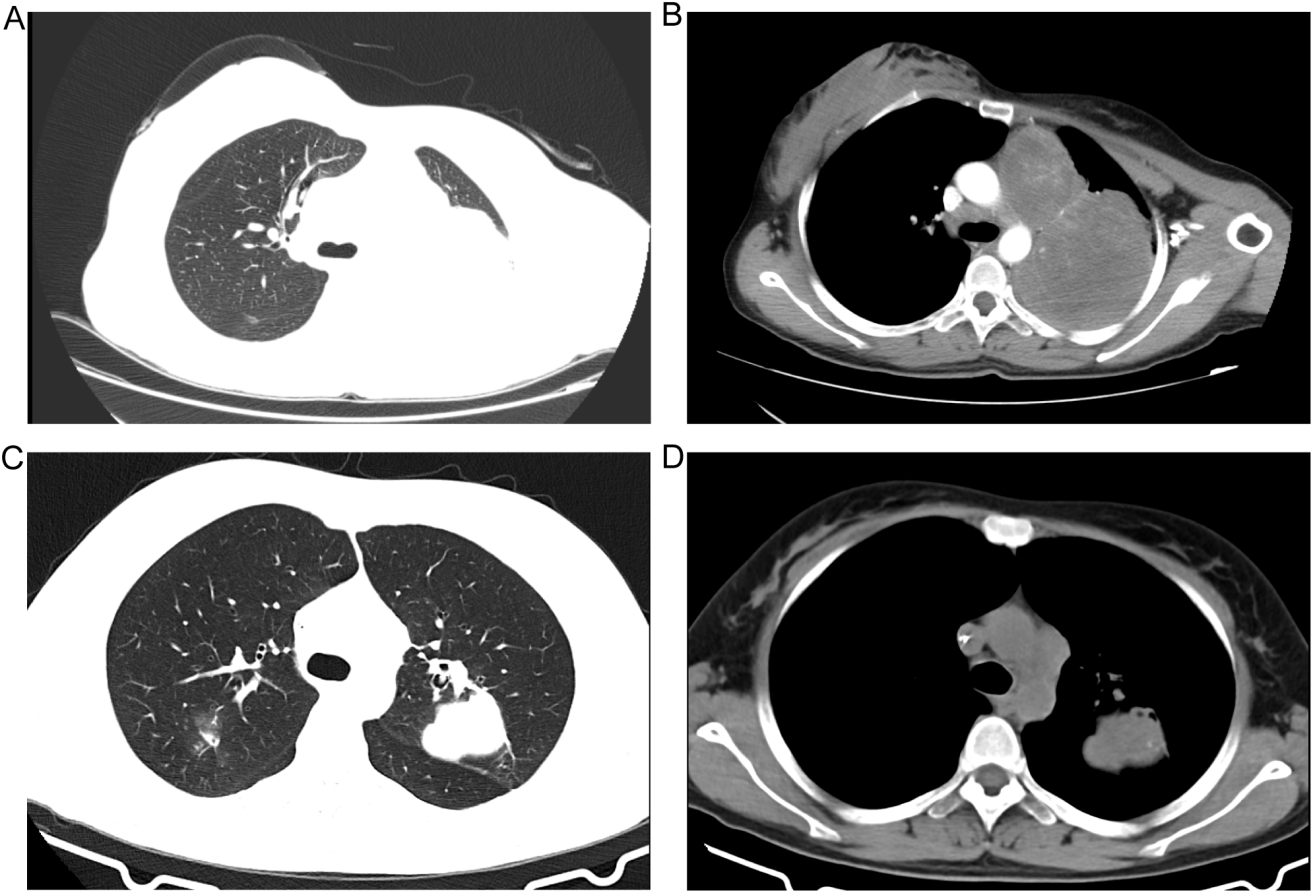
Comparison of chest CT scans before and after neoadjuvant chemotherapy. Pre-treatment contrast-enhanced CT scans showing the primary tumor in the **(A)** lung window and **(B)** mediastinal window. Post-treatment non-contrast CT scans after 6 cycles of neoadjuvant chemotherapy (ifosfamide + epirubicin regimen) showing tumor response in the **(C)** lung window and **(D)** mediastinal window.

A 32-year-old female presented with a 20-day history of cough, dyspnea, dizziness, and headache and was admitted on September 26, 2018. She had no significant medical history and was a nonsmoker. Physical examination revealed a palpable, hard, nontender, and moderately mobile enlarged left supraclavicular lymph node measuring approximately 4.7 × 2.8 cm. Her breath sounds diminished in the left lung. Contrast-enhanced chest CT revealed a large, well-defined, round-like mass (95 × 72 mm) in the left upper lobe, with widening of the left upper mediastinum. Enlarged lymph nodes were noted in the left supraclavicular region, aortopulmonary window, and bilateral hila. Two mass shadows with heterogeneous density (approximately 66 × 49 mm and 45 × 33 mm) were observed adjacent to the aortic arch and within the left upper lobe, containing speckled calcifications. These lesions showed mild to moderate enhancement with low-density areas and nodular enhancement upon contrast agent administration. The left upper lobe bronchus was compressed and narrowed (**Figures 1A, B**). The imaging diagnosis was a left lung neoplastic lesion with invasion of the mediastinal and hilar lymph nodes, accompanied by left lower lobe atelectasis and infection. Further investigations, including ultrasound of the liver, gallbladder, and spleen, adrenal CT, contrast-enhanced cranial MRI, and whole-body bone scintigraphy, revealed no distant metastases. Laboratory tests revealed a normal carcinoembryonic antigen (CEA) level. Alpha-fetoprotein (AFP) was elevated at 20.88 ng/mL (reference range 0–7 ng/mL), neuron-specific enolase (NSE) at 39.60 ng/mL (0–25 ng/mL), cytokeratin 19 fragment (CYFRA21-1) at 3.85 ng/mL (0–3.3 ng/mL), and lactate dehydrogenase (LDH) at 450 U/L (106–245 U/L). Percutaneous lung biopsy of the left upper lung lesion revealed a spindle cell malignant tumor, suggestive of biphasic synovial sarcoma. Biopsy of the left supraclavicular lymph node confirmed a metastatic malignant tumor. A left cervical lymph node biopsy was performed on September 28, 2018. Because the lung biopsy pathology indicated biphasic synovial sarcoma, a tumor with morphological overlap with CBPB, we chose a chemotherapy regimen targeting sarcoma. Chemotherapy with the IFO + EPI regimen (ifosfamide 2.8 g d1–5; epirubicin 95 mg d1–2) was initiated on October 12, supplemented with mesna rescue. Owing to the occurrence of grade IV bone marrow suppression after chemotherapy, the dose was adjusted to 90% of the original dose from November 2018 to March 2019 to complete the subsequent 5 cycles. After 6 cycles of chemotherapy, the response evaluation indicated a partial response (PR). The left upper lung mass had reduced to 60 × 50 mm, and the lesions adjacent to the aortic arch and within the left upper lobe had shrunk to 9.7 × 9.4 mm and 23 × 12 mm, respectively (**Figures 1C, D**). During chemotherapy, the AFP level continued to rise, peaking preoperatively at 821 ng/mL, whereas the LDH level normalized after the second cycle. Considering the completion of 6 chemotherapy cycles and the diminishing tumor reduction effect in the latter 2 cycles, a multidisciplinary discussion was held. Given that chemotherapy efficacy had reached a plateau and synovial sarcoma is relatively radioresistant, and considering that a large tumor volume would require extensive radiation fields with an increased risk of radiation injury, our multidisciplinary treatment (MDT) team recommended surgical debulking, particularly given the patient’s young age and strong treatment motivation.

**Figure 2:**
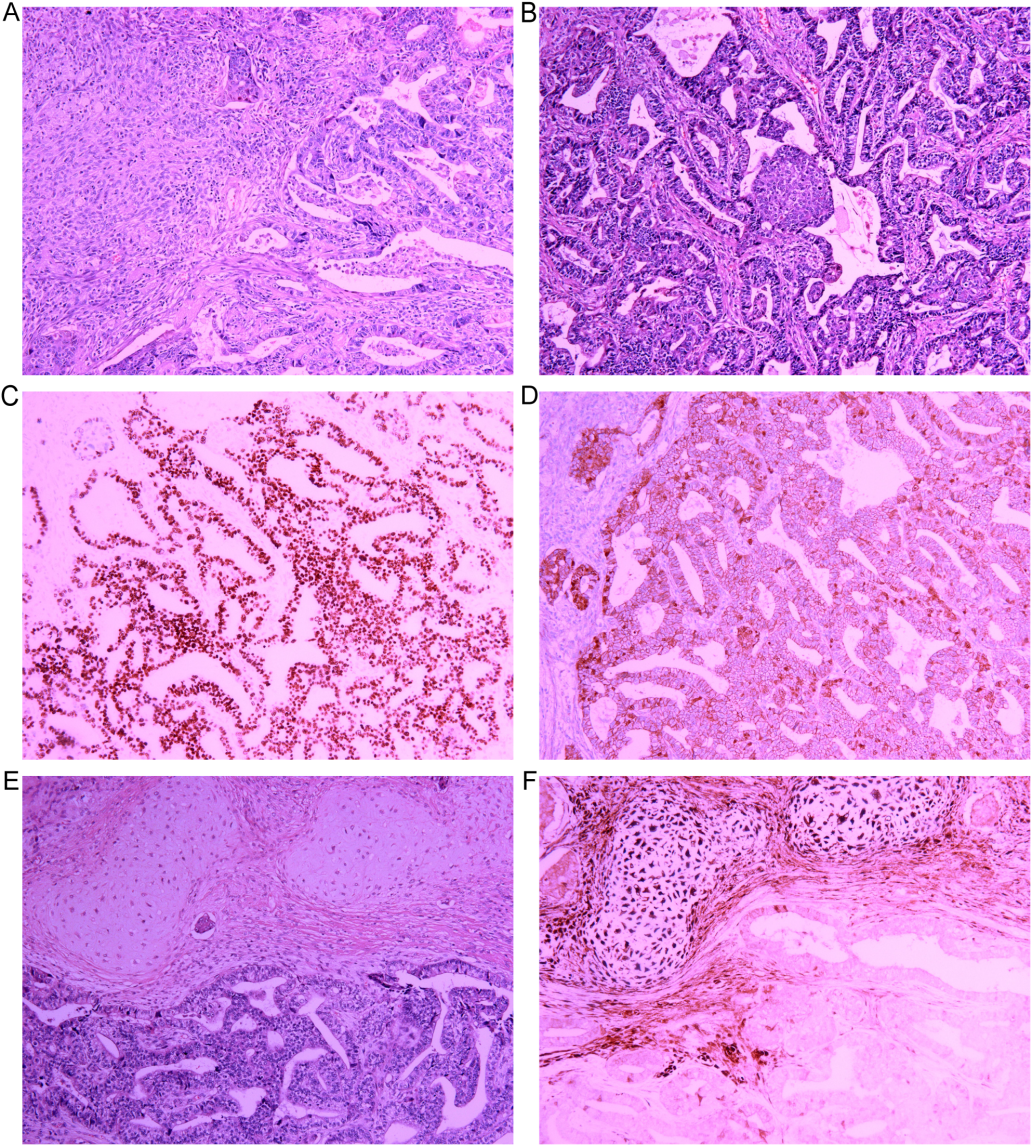
Histopathological and immunohistochemical features of the classic biphasic pulmonary blastoma patient. **(A)** Biphasic differentiation: The tumor is composed of varying proportions of epithelial (adenocarcinoma) and mesenchymal components. The mesenchymal cells exhibit a primitive blastomatous morphology with oval to short spindle shapes, arranged around glandular structures (H&E ×100). **(B)** Adenocarcinoma morphology: The epithelial component consists of well-differentiated fetal adenocarcinoma, characterized by complex, branching glandular structures lined by glycogen-rich, non-ciliated pseudostratified columnar cells with subnuclear vacuolization, resembling endometrial epithelium at low magnification. Focal mulberry-like structures are visible (H&E ×100). **(C)** Nuclear positivity for TTF-1 in tumor cells (original magnification ×100). **(D)** Aberrant β-catenin expression showing cytoplasmic/nuclear staining pattern (original magnification ×100). **(E)** Area of chondroid differentiation within the mesenchymal component (H&E ×100). **(F)** S-100 positivity in chondrocytes (original magnification ×100).

On March 29, 2019, the patient underwent left thoracic exploration, lysis of pleural adhesions, left upper lobectomy, and lymph node dissection under general anesthesia. Intraoperatively, the inferior margin of the mass was found invading the superior pulmonary vein. The enlarged mediastinal lymph nodes (groups 5 and 6) were densely adherent to the phrenic nerve, vagus nerve, and aortic arch, with superior extension invading the aortic arch. Owing to the risk of damaging critical structures with complete dissection, the lymph nodes above the pulmonary artery were cleared, while residual subaortic lymph nodes were left *in situ*. Postoperative recovery was uneventful. The final postoperative pathological diagnosis was pulmonary blastoma. Two lymph nodes from Group 6 exhibited spindle cell proliferation and coagulative necrosis, which is consistent with tumor metastasis. Bronchial margins and lymph nodes from Groups 7, 9, 10, and 11 were free of tumors. The immunohistochemistry results were as follows: CK (+), CR (−), Ki67 (labeling index <5%), Napsin A (+), TTF-1 (+), T-1 (positive in chondroid differentiation areas), S-100 (positive in chondroid differentiation areas), and SIA (negative in mesenchymal cells). For group 6 lymph nodes, CK (−), SIA (−), and β-catenin (+) were detected, with membranous staining in ~35% of adenocarcinomatous epithelial cells and cytoplasmic/nuclear staining in ~62% **Figure 2**. Genetic testing revealed a PIK3CA missense mutation, while EGFR, ALK, ROS1, BRAF, KRAS, ERBB2, RET, MET, and NRAS were all negative.

The surgery did not achieve complete tumor resection (R2 resection, macroscopic residual). To enhance tumor control, adjuvant chemoradiotherapy was planned postoperatively. Postoperative pathology showed a typical biphasic morphology (primitive epithelial glands surrounded by blastema-like spindle cells). Immunohistochemical positivity for TTF-1 and Napsin A supported a pulmonary origin, and aberrant β-catenin expression was more consistent with the features of CBPB than synovial sarcoma. Therefore, the final pathological diagnosis was revised from biphasic synovial sarcoma to CBPB. Considering the efficacy shown by the preoperative neoadjuvant chemotherapy regimen (IFO+EPI), and as this regimen is also commonly used for adjuvant therapy in CBPB, the original regimen was continued postoperatively.

Postoperative adjuvant chemotherapy (IFO + EP regimen) was started on May 17, 2019, but could not be completed due to adverse effects. From June 17 to July 31, 2019, the patient received concurrent chemoradiotherapy (radiation dose of 54 Gy combined with the DC regimen). Chemotherapy with the DP regimen (docetaxel 120 mg + cisplatin 110 mg) was administered on October 24 and November 23, 2019, completing the entire treatment course. The patient was followed up regularly, and no tumor recurrence was observed at follow-up as of July 2025.

## Discussion

The clinical presentation of PB is not significantly different from that of common non-small cell lung cancers. Patients often present with symptoms such as cough (33%), hemoptysis (29%), and chest pain (33%) (8). The etiology of PB remains unclear. While some reports suggest a potential risk factor for smoking, definitive causative factors require further exploration (5, 6). Radiographic features of PB typically include a solitary, peripheral, round or oval soft tissue mass with relatively homogeneous density, often larger than 5 cm in diameter (average 7–10 cm). Lesions are more frequently located in the upper lobes and may contain calcifications. Contrast-enhanced scans revealed mild to moderate enhancement. The mass may exhibit large vessel traversing and can be associated with atelectasis, obstructive pneumonia, or pleural effusion (11–14).

Historically, PB has been classified into three subtypes on the basis of age of onset and pathological features: pleuropulmonary blastoma (PPB), classic biphasic pulmonary blastoma (CBPB), and well-differentiated fetal adenocarcinoma (WDFA) (3). The 2021 World Health Organization (WHO) Classification of Thoracic Tumors introduced significant revisions, clearly distinguishing these as separate disease entities: CBPB was reclassified as a sarcomatoid carcinoma, WDFA was categorized under adenocarcinomas, and PPB was classified as a soft tissue tumor (7). Owing to its biphasic differentiation ability, CBPB is characterized by the immunohistochemical expression of both epithelial markers (e.g., CK, CEA, NSE, CgA, and EMA) and mesenchymal markers (e.g., vimentin, SMA, desmin, and S-100) (3, 15). Molecular profiling of PB revealed that genetic alterations involve primarily the CTNNB1 (β-catenin) and DICER1 genes. Sekine et al. (16) identified somatic mutations in CTNNB1, specifically affecting exon 3, in all WDFAs and a subset of CBPBs. More recently, Tian et al. (17) confirmed a high frequency of CTNNB1 exon 3 mutations (71.4%) and reported that DICER1 alterations occur in up to 86% of PB cases, often following a “two-hit” mode, underscoring their central role in PB pathogenesis.

CBPB has traditionally been considered a highly aggressive tumor with an overall poor patient prognosis. Previous clinical observations have indicated that approximately two-thirds of patients die within 2 years of diagnosis, with 5-year and 10-year overall survival rates as low as 16% and 8%, respectively (8). A pooled analysis of 66 CBPB patients reported between 2000 and 2022 by Yao et al. (2) also revealed a median survival of only 1 year and an approximate 5-year survival rate of 9%. However, a SEER database study published in 2024, which strictly adhered to the new 2021 WHO classification and analyzed 65 PB patients diagnosed between 2000 and 2020, reported significantly better survival outcomes: the 1-year, 3-year, and 5-year overall survival rates were 70.3%, 57.2%, and 51.9%, respectively (4). This marked improvement in survival rates may be attributed to several factors, including the refinement of case definitions (the new criteria excluded prognostically poorer PPB), a high rate of surgical intervention (73.85% in this study), and overall advances in medical care. This suggests that historical data may have systematically underestimated the existence of a subgroup of CBPB patients who can achieve long-term survival through aggressive multimodal therapy.

To further elucidate the factors associated with long-term survival, this study defined CBPB patients who survived more than 3 years as “long-term survivors.” Given the multiple updates to the AJCC TNM staging system during this period, we uniformly restaged all included cases according to the 9th edition criteria. We focused on their clinical characteristics and treatment strategies before and after recurrence, aiming to identify key factors potentially improving the prognosis of successfully managed patients. A literature search was performed in the PubMed and Web of Science databases for studies published from 2000 to the present using the following search query: (“Pulmonary Blastoma*” OR “Blastoma*, Pulmonary”) NOT (“pleuropulmonary blastoma*”). The inclusion criteria were adult patients (≥18 years) with pathologically confirmed CBPB and survival >3 years reported in the English literature. Exclusion criteria included pediatric cases (<18 years), reviews, and reports with incomplete data. Two investigators independently performed screening, full-text review, and data extraction, with discrepancies resolved through discussion. As most included studies were case reports, no formal quality assessment was performed, but data completeness and consistency were evaluated. Twelve articles (encompassing 13 patients) were ultimately included for further analysis.

As shown in **Table 1**, among the 13 analyzed long-term survival CBPB patients, 38.46% (5/13) survived ≥ 5 years, and 15.38% (2/13) survived ≥10 years. Patients were predominantly young adults (aged < 65 years), accounting for 69.23% (9/13) of the sample. The tumors were generally large, with minimum diameters of ≥5 cm (ranging up to 16 cm) and an average diameter of 9 cm. Staging was predominantly stage III (76.92%, 10/13), whereas stage II and stage IV accounted for 15.38% (2/13) and 7.69% (1/13), respectively. Notably, the vast majority of patients (69.23%, 9/13) had no lymph node metastasis (N0 stage) at initial diagnosis. Twelve patients (92.3%, 12/13) underwent surgical resection of the primary lung lesion. Among these patients, 50% (6/12) received postoperative adjuvant chemotherapy, and three received radiotherapy. Among the 9 patients who experienced recurrence, 4 underwent repeat resection of metastatic lesions. In particular, in patients surviving more than 5 years, the repeat resection rate after recurrence is as high as 60%, often combined with adjuvant radiotherapy and/or chemotherapy. In addition, two patients demonstrated marked sensitivity to antiangiogenic agents (anlotinib and sorafenib). Another patient with unresectable stage IIIB disease who progressed after chemoradiotherapy received anti-PD-1 therapy (sintilimab) and achieved a progression-free survival of > 27 months and an overall survival exceeding 40 months. These findings suggest that antiangiogenic therapy and immunotherapy hold considerable potential in the treatment of CBPB.

**Table 1:** Overview of long-lived cases of biphasic pulmonary blastoma from 2000 to 2025.

Pat N0.	Age(years)	Sex	Smoking	Size (cm)	Lobe	Stage	Stage migration(ADJJ 9)	surgery	CT	RT	Recurrence time (months)	Metastasis	Treatment after recurrence	Outcome	Refs.
														(months)	
1	58	M	Y	5	RUL	T3N0M0	PT3N0M0 IIB	Lob + lym	Pac, Ned	NA	24	Liver, Lung	CT (Ned, Pac, EP, Pem, Carbo, Bev) + Anlotinib	Alive wr 48	(28)
2	71	F	Y	7	RLL	T2N0M0	PT4N0M0 IIIA	Lob + lym	NA	NA	23	Vertebra, Adrenal gland	Surgery + CT (PDD, VP-16, Carbo) + RT (30GY)	Alive wr 84	(35)
3	38	F	Y	10	LUL	T3N2M0	pT4N2M0 IIIB	Lob + lym	PDD, Vin, Doc	50.4 Gy	?	Brain	Surgery + RT (WBRT)	Alive wr 120	(19)
4	29	F	N	9	LLL	T4N1M1	pT4N1Mx	Lob + lym	NA	NA	2	Ovary	Surgery + CT (PDD, If, VP-16) + RT(59.49 Gy)	Alive wr 120	(19)
5	73	F	?	5	RUL	T2N0M0	PT2bN0M0 IIA	Lob	NA	NA	NA	NA	NA	Alive wr 48	(8)
6	70	M	?	7	RUL	T3N0M0	PT4N0M0 IIIA	Lob + lym	NA	NA	NA	NA	NA	Alive wr 36	(36)
7	47	F	N	15	LUL	T4N0M0	PT4N0M0 IIIA	Pne	VP-16, Carbo, If	60 Gy	NA	NA	NA	Alive wr 36	(37)
8	58	M	?	10	RUL	T4N0M0	PT4N0M0 IIIA	Lob + lym	PDD, VP-16	NA	27	Chest wall	RT (50 Gy)	Alive wr 70	(38)
9	73	M	?	7	RLL	T2N0M0	PT4N0M0 IIIA	Lob + lym	Exist	NA	NA	NA	NA	Alive wr 36	(39)
10	37	F	?	9	RUL	T4N?M0	pT4N0M0 IIIA	Lob + lym	Gem, Carbo	NA	4	Chest wall, Kidney	Surgery (Chest wall*2 + Kidney)+ CT (Pem, Bev, Sor) + RT (48Gy)	Alive wr 36	(20)
11	48	M	Y	16	RUL	T4N0M0	pT4N0M0 IIIA	Lob	EPI, IFO	NA	18	Lung	CT (GEM, Carbo, NVB)	DOD 41	(40)
12	43	M	?	8	RLL	T4N2M0	PT4N2M0 IIIB	Lob+ lym	NA	NA	1	Lung	CT (DOC, PDD)	Alive wr84	(41)
13	50	M	Y	10	LUL	T4N2M0	CT4N2M0 IIIB	NA	Pac, PDD	75Gy	12	Lung	CT(GEM)+ Sintilimab	Alive wr40	(42)

Alive wr, alive without recurrence; Bev, bevacizumab; Carbo, carboplatin; CT, chemotherapy; Doc, docetaxel; DOD, death of disease; Epi, epirubicin; Gem, gemcitabine; If, ifosfamide; LLL, left lower lobe; lob, lobectomy; LUL, left upper lobe; lym, lymphadenectomy; Ned, nedaplatin; Pac, paclitaxel; PDD, cisplatin; Pem, pemetrexed; pne, pneumonectomy; RLL, right lower lobe; RT, radiotherapy; RUL, right upper lobe; Sor, sorafenib; Vin, vinorelbine; VP-16, etoposide;NVB, Vinorelbine; NA, Not Applicable.

Since its initial description in 1945, surgical resection has remained the cornerstone of PB treatment. The survival difference between surgical and nonsurgical patients is substantial. As early as 1991, Koss and colleagues reported that the median overall survival (OS) was only 2 months in patients who did not undergo resection, whereas it extended to 33 months in those who did (18). Similarly, Zaidi et al. reported a median OS of 43.5 months in four surgically treated patients versus 5.5 months in two patients managed without surgery (6). Among the 13 long-term surviving patients, 12 (92.3%) underwent complete resection, underscoring the central role of surgery in the multimodal management of CBPB.

Furthermore, aggressive local intervention may also confer survival benefits in cases of recurrence and metastasis. Our study revealed that half of the long-term survivors underwent repeat resection after recurrence. This strategy is supported by the literature. For example, Lewis JA et al. reported two CBPB cases: one patient with brain metastasis achieved long-term survival after multimodal therapy, including craniotomy; another patient developed pelvic metastasis after chemotherapy and survived more than 10 years following metastasectomy (19). Additionally, Mulamalla K et al. described a patient who, after experiencing chest wall recurrence and right renal metastasis, achieved a total survival time exceeding 3 years through repeat resection combined with sorafenib-targeted therapy (20). These cases indicate that selected patients with recurrent or metastatic CBPB can achieve durable remission through aggressive multidisciplinary therapy, especially repeat resection.

Currently, there is no standardized chemotherapy regimen for CBPB. Larsen and Sorensen reported an overall response rate of 26% to chemotherapy among 43 PB patients, with efficacy significantly influenced by tumor heterogeneity (21). The value of neoadjuvant chemotherapy in locally advanced PB remains inconclusive, yet studies have demonstrated its potential. Zaidi et al. (6) reported two stage IIIb patients who were successfully downstaged with neoadjuvant chemotherapy and subsequently underwent surgery, achieving survival times of 35 and 29 months. Bosch-Barrera J et al. (22) described a 25-year-old patient with stage III CBPB who successfully underwent surgery after induction chemoradiotherapy, resulting in favorable survival outcomes. Similarly, the present stage IIIc CBPB patient achieved tumor shrinkage and downstaging through neoadjuvant chemotherapy. Although R0 resection was not performed, subsequent aggressive chemoradiotherapy ultimately led to long-term survival and a clinical cure. Given the remarkable survival difference between surgical and nonsurgical management, for patients with locally advanced disease, efforts should be made to create surgical opportunities via neoadjuvant therapy (chemotherapy or chemoradiotherapy), particularly for young patients with good performance status.

In recent years, targeted therapy and immune checkpoint inhibitors (ICIs) have fundamentally reshaped the therapeutic landscape of lung cancer. Although research on targeted PB therapy is scarce, small-scale retrospective analyses have revealed several potential targets. Macher-Goeppinger S et al. (23) reported EGFR expression in all five PB patients and a CTNNB1 mutation in three of five patients, suggesting potential therapeutic value for EGFR inhibitors. These molecular findings support the role of CTNNB1 mutations in PB pathogenesis and indicate their diagnostic utility in distinguishing PB from other lung cancers. Meng et al. (24) reported a case of stage IV CBPB with a *CD74-ROS1* rearrangement that achieved a rapid partial response to crizotinib, providing the first confirmation of the efficacy of targeted therapy against specific driver genes in CBPB. Another study (25), which performed targeted sequencing on 16 patients, preliminarily delineated the genetic mutation profile of PB, encompassing genes such as BRCA2, ERBB4, and ALK. These findings indicate the clinical potential of precision therapy on the basis of molecular characteristics, although its efficacy requires further validation.

Aberrant angiogenesis is a hallmark of the tumor microenvironment. Studies have shown that PBs are highly vascularized and that angiogenesis promotes the proliferation, invasion, and metastasis of PBs (26), providing a rationale for antiangiogenic therapy. Bevacizumab, an anti-VEGF monoclonal antibody, exerts its antitumor effect by inhibiting tumor angiogenesis (27). Several case reports support the efficacy of this strategy. For example, Luo et al. (28) reported a patient with recurrent CBPB who achieved long-term disease control with oral anlotinib after various chemotherapy regimens, including bevacizumab. Mulamalla K et al. (20) reported a patient who responded to sorafenib, enabling subsequent metastasectomy.

ICIs represent one of the most successful forms of cancer immunotherapy, primarily including programmed cell death protein-1 (PD-1) and its ligand PD-L1 inhibitors, as well as cytotoxic T lymphocyte-associated protein 4 (CTLA-4) inhibitors (29). The mechanism involves blocking tumor-induced suppression of T-cell function, thereby reactivating antitumor immune responses. PD-L1 has been established as a classic predictive biomarker for immunotherapy efficacy and has been validated in several pivotal clinical trials (30, 31). In 2015, Bosch-Barrera et al. first reported a case of unresectable locally advanced CBPB with high PD-L1 expression (>90% of tumor cells), suggesting that PD-1/PD-L1 inhibitors could offer a new immunotherapeutic option for this rare malignancy with a poor prognosis (22). More recently, a stage IIIA PB patient was treated with the PD-1 inhibitor penpulimab combined with the multitarget tyrosine kinase inhibitor anlotinib, resulting in significant tumor shrinkage. The patient achieved a major pathologic response after surgery and received postoperative maintenance immunotherapy (32). Integrated molecular characterization of PB has further revealed that although PB generally has a low tumor mutation burden and leukocyte fraction, some cases show focal PD-L1 positivity or CD8+ T-cell infiltration, supporting the potential applicability of immunotherapy in selected patients (17).While case reports suggest that antiangiogenic agents and immunotherapy may be effective in some CBPB patients, the overall evidence base remains extremely weak. The rationale for these strategies derives from the angiogenic characteristics of CBPB and the potentially active immune microenvironment (e.g., PD-L1 expression) (17, 32–34). Given that their efficacy is far from established, these therapies should currently be considered only as exploratory options after standard treatment failure or within strictly designed clinical studies. Defining their value requires future research incorporating molecular biomarkers to identify potential responders. Although evidence primarily stems from case reports, antiangiogenic agents undoubtedly represent a promising new option for CBPB patients, especially those for whom conventional therapies have failed.

## Conclusion

This case demonstrates that a multimodal therapeutic paradigm comprising neoadjuvant chemotherapy, surgical resection, and postoperative radiotherapy can enable long-term disease-free survival and even a clinical cure in select patients with stage IIIC CBPB. Integrated with literature analysis, radical surgery forms the cornerstone for achieving long-term survival, whereas aggressive resection for recurrent or metastatic lesions represents a critical strategy. However, this study is a single-center case report, and conclusions should be extrapolated cautiously. Additionally, the literature review included heterogeneous cases, most lacking systematic molecular data. Future directions should focus on establishing prospective registries through multicenter collaboration to accumulate high-level evidence. Meanwhile, the value of immunotherapy, targeted therapy, and antiangiogenic agents in precision populations warrants further exploration.

## Data Availability

The original contributions presented in the study are included in the article/Supplementary Material. Further inquiries can be directed to the corresponding author.
